# Roles for c-Abl in postoperative neurodegeneration

**DOI:** 10.7150/ijms.73740

**Published:** 2022-09-28

**Authors:** Long Feng, Shihui Fu, Yao Yao, Yulong Li, Longhe Xu, Yali Zhao, Leiming Luo

**Affiliations:** 1 Department of Anesthesiology, Hainan Hospital of Chinese People's Liberation Army General Hospital, Sanya, China.; 2 Department of Cardiology, Hainan Hospital of Chinese People's Liberation Army General Hospital, Sanya, China.; 3 Department of Geriatric Cardiology, Chinese People's Liberation Army General Hospital, Beijing, China.; 4 Center for the Study of Aging and Human Development and Geriatrics Division, Medical School of Duke University, North Carolina, USA.; 5 Center for Healthy Aging and Development Studies, National School of Development, Peking University, Beijing, China.; 6 Department of Anesthesiology, The Third Medical Center of Chinese People's Liberation Army General Hospital, Beijing, China.; 7 Central Laboratory, Hainan Hospital of Chinese People's Liberation Army General Hospital, Sanya, China.

**Keywords:** Alzheimer's disease, Nonreceptor tyrosine kinase, Oxidative stress, Parkinson's disease, Postoperative cognitive dysfunction, Postoperative neurodegeneration

## Abstract

The nonreceptor tyrosine kinase c-Abl is inactive under normal conditions. Upon activation, c-Abl regulates signaling pathways related to cytoskeletal reorganization. It plays a vital role in modulating cell protrusion, cell migration, morphogenesis, adhesion, endocytosis and phagocytosis. A large number of studies have also found that abnormally activated c-Abl plays an important role in a variety of pathologies, including various inflammatory diseases and neurodegenerative diseases. c-Abl also plays a crucial role in neurodevelopment and neurodegenerative diseases, mainly through mechanisms such as neuroinflammation, oxidative stress (OS), and Tau protein phosphorylation. Inhibiting expression or activity of this kinase has certain neuroprotective and anti-inflammatory effects and can also improve cognition and behavior. Blockers of this kinase may have good preventive and treatment effects on neurodegenerative diseases. Cognitive dysfunction after anesthesia is also closely related to the abovementioned mechanisms. We infer that alterations in the expression and activity of c-Abl may underlie postoperative cognitive dysfunction (POCD). This article summarizes the current understanding and research progress on the mechanisms by which c-Abl may be related to postoperative neurodegeneration.

## Background

Neurodegenerative diseases, such as Alzheimer's disease (AD), Parkinson's disease (PD), amyotrophic lateral sclerosis (ALS) and frontotemporal dementia, are common central nervous system (CNS) diseases 1-5. These diseases lead to the gradual impairment of CNS functions, such as activity, memory, learning, judgement and coordination. Besides, it often leads to postoperative cognitive dysfunction (POCD) after surgery with anesthesia in the elderly, which is more common after cardiac surgery than other types of surgery 6. Developmental neurotoxicity induced by general anesthetics can cause acute widespread neuronal cell death affecting long-term memory and learning defects of the infants and young children 7-8. The incidence of POCD decreases over time, with the rate being the highest (30% to 70%) at hospital discharge, 12% to 21% 3 months after anesthesia and noncardiac surgery, 20% to 30% 6 months after surgery, and 15% to 25% after 12 months of follow-up 9-13. POCD can prolongs hospitalization and rehabilitation time, increases the incidence of disability, and decreases life quality and survival, thus resulting in a heavy economic burden on the health care system 5,14-15. Compared with common neurodegenerative diseases and POCD, not only do they share the same characteristic symptoms, at the same time brain structural changes are similar, including cerebral white matter changes, central neuroinflammation, neuronal apoptosis, and so on 16-20.

Brain aging is a complex process that affects everything from the subcellular level to the organ level, starting early in life and accelerating with aging process 21. Morphologically, brain aging is mainly characterized by brain volume loss, ventricular enlargement, cortical thinning, vitrification loss and white matter degradation. Pathophysiologically, brain aging is associated with neuron atrophy, dendritic degeneration, metabolic slowing, microglial activation, demyelinating disease, small vessel disease, and formation of white matter lesions (WML) 21. WML is caused by cerebral hypoperfusion, observed in the elderly, related to cognitive decline, and prevalent in AD patients 22. Multiple sclerosis (MS) is a chronic inflammatory demyelinating disease of CNS that causes focal VML and diffuse neurodegeneration throughout the brain. Range of MS lesions is in relation to inflammatory processes 23. Furthermore, apoptosis is a programmed cell death that plays a key role in nervous system development and chronic neurodegenerative diseases, including AD and MS 24-26. Neuroinflammation and neurodegeneration were caused by surgery and general anesthesia. Patients with POCD had significantly more white matter lesions and greater gray matter loss (medial temporal lobe) 27. Different anesthetics and neuroinflammation will cause the apoptosis of neurons and increase the incidence of POCD 28-30.

c-Abl was first discovered in Aberson murine leukemia virus by Ozanne et al. in 1982 31. c-Abl (ABL, Abl1) and Abelson (ABL)-related genes (Arg, Abl2) have been identified as members of the c-Abl family of tyrosine kinases. c-Abl family members are highly conserved among different species and are involved in many cell regulatory processes, including regulation of actin cytoskeleton, cell cycle, stress-induced apoptosis and cycle arrest 32. Under normal circumstances, apoptosis is an important physiological process that maintains the stability of internal environment and ensures normal tissue development and growth. Previous research has found that activated c-Abl can participate in apoptosis by interacting with multiple factors 33. The gene is inactive under normal conditions. Upon activated and overexpression, this kinase can cause cell apoptosis, cycle alterations and pathological changes that may be related to neurodegenerative diseases 32-40. Other studies have shown that Abl family kinases, especially c-Abl, play a critical role in neurodevelopmental and neurodegenerative diseases. Selective inhibition of c-Abl expression and activity has neuroprotective effects 32-39. In addition, abnormally activated c-Abl is associated with a variety of neurodegenerative diseases. In studies using AD and PD models, it was found that c-Abl inhibitors can promote amyloid clearance and reduce the neural inflammation, which are two key drivers of nerve cell death 41. Therefore, a large amount of literature has proposed that this kinase is crucial in regulating neurodegenerative diseases. The main mechanism of anesthesia-induced POCD is very similar to the mechanism by which c-Abl is involved in the pathogenesis of neurodegenerative diseases.

c-Abl activation regulates neuronal death response to Aβ fibrils. Intraperitoneal administration of imatinib rescued cognitive decline, Tau phosphorylation, and caspase-3 activation in neurons surrounding Aβ deposits 42. Imatinib reduces plasma β-Oligomers and brain features, such as Oligomer accumulation, neural inflammation and cognitive deficits. The results support the role of c-Abl in Aβ accumulation of neurodegenerative diseases, and the efficacy of imatinib in the treatment of these diseases 43. Besides, the c-Abl-drp1 signaling pathway regulates oxidative stress-induced mitochondrial fragmentation and cell death, which may be a potential target for the treatment of neurodegenerative diseases 44. Furthermore, previous studies have found that intravenous anesthetic propofol significantly reduces c-Abl expression, but reducing c-Abl expression by propofol did not impair learning or memory function 45. This study illustrates the involvement of c-Abl in the POCD process.

**Figure 1:**
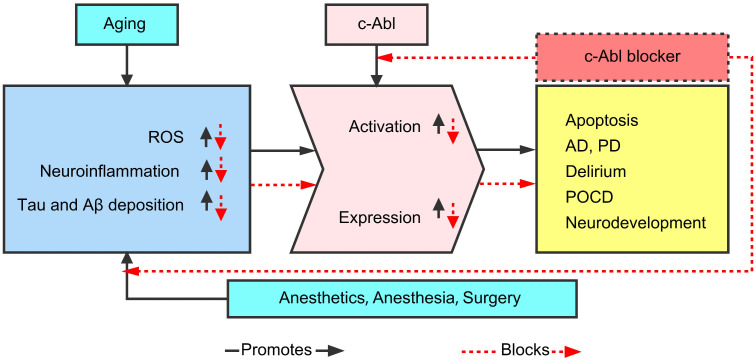
The current understanding and research progress on the mechanisms by which c-Abl may be related to neurodegenerative diseases.

Therefore, this paper hypothesized that the expression and activation of c-Abl are abnormally elevated in various neurodegenerative diseases (AD, PD, ALS, MS, POCD, etc.), mainly as a result of abnormality in the Tau and Aβ proteins caused by neuroinflammation and oxidative stress (OS). In addition, various c-Abl blockers (imatinib, nilotinib, dasatinib, ladotinib and other drugs) can effectively reduce abnormal proteins and ameliorate cognitive dysfunction. As shown in Figure 1, this article summarizes the current understanding and research progress on the mechanisms by which c-Abl may be related to neurodegenerative diseases.

## Neuronal cell development

ABL and Arg tyrosine kinases play an important role in the development and function of neuronal systems. Abl1 and Abl2 are downstream targets of ABL family to regulate cell growth and transformation. They may have unique and common functions in the development of CNS. Changed phosphorylation and molecular weight of ABL protein that occur during the maturation of CNS suggest that Abl1 and Abl2 may be involved in the signaling events that are responsible for regulating neural cell development 46. Koleske and others found that ABL and Arg kinases are expressed at the highest levels in the neuroepithelium at Embryos day9 (E9) 47. Arg is more abundant in the adult mouse brain, especially in the synaptic-rich regions. Arg deficient mice develop normally but show several behavioral abnormalities, indicating the existence of brain defects in these mice 47. Embryos lacking both ABL and Arg develop neurodevelopmental defects 47. ABL kinase-mediated signal transduction from different cell surface receptors also regulates cell proliferation and survival during cell development and homeostasis 48. In addition, during normal axonal development, axonal growth is promoted by the binding of kinesin-1 to c-Abl and their interaction. The c-Jun interacting protein-1 (JIP1) is an important regulator of axonal development and a key target of c-Abl-dependent pathway in controlling axonal growth 49. In addition, during neurite growth, c-Abl binds to and activates cyclin-dependent kinase 5 (CDK5), affecting neuronal migration and growth 35. In addition to its typical functions in the pathogenesis of leukemia, c-Abl is also considered to play an important role in neuronal development, neuronal migration, axon extension and synaptic plasticity 37-39. Miller et al found that c-Abl and ataxia-telangiectasia mutation (ATM) are very important for development and survival, especially after genotoxic stress, and that they have obvious selectivity for the developing nervous system 50. In addition, c-Abl functionally interacts with p53 during cell development, and mice lacking them are unlikely to be viable 51. Therefore, c-Abl is associated with a variety of cellular processes, including the regulation of cell growth and survival, as c-Abl deficient mice are embryonic or neonatal lethal 52,53.

## Neurodegenerative diseases

A large number of studies have found that c-Abl is activated in human neurodegenerative diseases, especially AD 32-41. Bowser's team found that the phosphorylation of c-Abl at Y412 and granulovacuolar degeneration (GVD) in the brain are common in patients with AD 54. Some studies have also found in PD that c-Abl expression is increased in the striatum and that the tyrosine phosphorylation is also increased 55. However, the pathogenesis of AD and POCD is still unclear. The possible mechanisms underlying the diseases may involve the following processes. First, some scholars believe that amyloid cascade leads to the formation of Aβ fibrils. Increased aggregation of these fibrils leads to neuroinflammation, which in turn alters the physiology of neurons and induces oxidative stress in these cells, ultimately leading to kinase activation, the formation of tangles and reduced nerve cells. In addition, a study suggested that the mechanism of AD may be related to an abnormal neuroinflammatory response caused by initial injury 56. The signaling pathway activated by c-Abl may be related to growth factors, cell adhesion and OS 46-47.

## Oxidative stress

Upon aging and disease development, the body's ability to deal with OS and deoxyribonucleic acid (DNA) damage during normal cell processes is weakened, resulting in the accumulation of oxygen free radicals and DNA damage. OS can regulate neurodegeneration through various mechanisms, including protein and lipid-related processes, Aβ deposition, cytokine production, mitochondrial dysfunction, proteasome dysfunction, and the formation of advanced glycation products, oxidation of nucleic acids and activation of glial cells 47. c-Abl generally exists in cells in inactive and activated states, with the activation of c-Abl being tightly regulated by intramolecular bonds. c-Abl protein complexes can also be linked to the membrane through an amino-terminal myristoyl group. Reactive oxygen species (ROS) may activate c-Abl by triggering ATM kinase activation through OS. In addition, a subtype of protein kinase C can activate and phosphorylate c-Abl under hydrogen peroxide (H_2_O_2_) stimulation 41,46,49. Some authors even believe that ROS can directly activate c-Abl 40. c-Abl can eventually cause cell dysfunction through DNA damage and increase the level of free radicals under stress conditions 32. Alvarez et al. found that c-Abl expression is upregulated in response to OS and the presence of Aβ fibrils in cultured neurons 57. In addition, c-Abl can be activated under OS, dopaminergic stress, and genotoxic stress, and exposure to these stresses leads to reduced and destructed neuronal cells 58-59. Therefore, OS is considered a potential therapeutic target for the prevention and treatment of neurodegenerative diseases through regulation of oxygen free radicals or alleviation of their harmful effects 60.

## Tau protein phosphorylation

Tau is a microtubule-associated protein and a major component of neurofibrillary tangles (NFTs). It is normally involved in the formation of microtubules and cytoskeletal dynamics, and abnormal phosphorylation of Tau protein can cause microtubule entanglement and instability. Hyperphosphorylation of wild-type Tau protein results in the formation of NFTs, which is one of the hallmark pathologies of individuals with AD 61. Excessive accumulation of toxic Tau protein can cause death and dysfunction of nerve cells and glial cells, thereby causing disease symptoms. Studies have shown that the exposure of cultured neuronal cells to various Aβ peptides can activate tyrosine kinases, in turn causing tyrosine phosphorylation of Tau protein. The E3 ubiquitin ligase is a common player to play a neuroprotective role in AD and PD through scavenging misfolded proteins, such as Aβ peptides and phosphorylated Tau protein 41. However, during OS response in neurodegenerative diseases, ABL translocates to mitochondria, where it phosphorylates Parkin, causing activity loss of E3 ubiquitin ligase. This causes abnormal accumulation of Aβ peptides and Tau protein and is responsible for neuronal apoptosis and cognitive dysfunction 41. Furthermore, previous studies have found that oligomeric Aβ peptides are present in the brain cells of AD patients during the induction of Tau protein hyperphosphorylation 62. In addition, Derkinderen and colleagues pointed out that c-Abl phosphorylates Tau protein at Y394 63. It was also found in AD brain that activated ABL is present in granular structures of hippocampal neurons.

## Neuroinflammation

Inflammatory mediators are detected in brain sections from AD and PD patients, and neuroinflammation may be one of the causes of these neurodegenerative diseases 64. Numerous studies have also found that the etiology of AD and PD may be related to chronic neuroinflammation 65-66. In the study of two transgenic mice (AblPP/tTA mice and ArgPP/tTA mice), it was found that c-Abl overexpression can lead to neuronal loss and neuroinflammatory response 65. Neuroinflammation can be triggered by a variety of biological mechanisms, including OS and glial response. Neuroinflammatory mediators, such as cytokines and prostaglandins, play an important role in the development of neurodegenerative diseases 67-69. In the early stage of neuroinflammation in AD, a vicious cycle of microglial activation, proinflammatory factor release and neuronal damage may occur 70. Some authors have pointed out that c-Abl activation by neuronal cells can lead to neurodegenerative changes and neuroinflammatory changes 66. Treatment of AD model mice with a c-Abl blocker led to the clearance of Aβ peptides, reduced the number of astrocytes and dendritic cells, and regulated the distribution of cytokines and chemokines 71. It has also been indicated that continuous overexpression of c-Abl in neurons can cause the degeneration of neuronal cells in the hippocampal region. In a follow-up study, it was found that this pathophysiological process is mainly caused by transient and obvious changes in the cell cycle that are associated with protein and DNA replication in the olfactory bulb and activation of transcription 1 (STAT1) signaling pathway, which is crucial for the regulation of c-Abl-induced neuroinflammation and neurodegenerative diseases 66, in the hippocampal region. Wu et al. found that c-Abl can abnormally activate P38 and lead to neuronal death in the neuroinflammatory environment 72. It is known that P38 is a member of mitogen activated protein kinases (MAPKs) family and plays an important role in inflammation, neurodegeneration and cell death. Therefore, according to all above these findings, we speculate that the activation of c-Abl in neurons leads to pathological changes related to neuroinflammation.

## Roles of c-Abl blockers

Imatinib, nilotinib, and bosutinib have been shown to inhibit proinflammatory cytokines (TNF-α, IL-1β, IL-6, iNOS, COX-2, NLRP3) production, thereby reducing the recruitment of inflammatory cells to central nervous system 71-74. AD is the most common neurodegenerative disease and the main cause of dementia. Its main neuropathological hallmarks are mainly the accumulation of extracellular neurotrophic plaques comprising Aβ peptides and the formation of intracellular and extracellular NFTs in brain regions. Neuroinflammation also plays an important role in the pathogenesis and progression of AD 74. Infection, trauma, ischemia, and toxins increase levels of proinflammatory cytokines, including TNF-α, IL-1β, IL-6, IL-18, and chemokines such as C-C motif chemokine ligand 1 (CCL1), CCL5 and C-X-C motif chemokine ligand 1 (CXCL1). The release of pro-inflammatory molecules can lead to synaptic dysfunction, neuronal death and neurogenesis inhibition. During the progression of AD, memory loss and cognitive decline are accompanied by the degradation of specific neurons in the hippocampus and cerebral cortex. Studies have shown that the phosphorylation of tyrosine kinases at T412 is significantly increased in the hippocampus and entorhinal cortex in the brains of AD patients 75. Moreover, Aβ can enhance the activity of c-Abl, and this kinase is involved in regulating the death of neurons 57. c-Abl blockers have also been shown to significantly reduce Tau protein phosphorylation in transgenic AD animal models. It has also been found *in vitro* that Aβ can significantly increase c-Abl expression and phosphorylation of Tau protein in nerve cells 76. This indicates that the expression of c-Abl is significantly increased during the pathogenesis of AD and that kinase blockers can significantly reduce the accumulation and expression of abnormal proteins.

In addition, similar studies have also shown that c-Abl blocker imatinib can significantly reduce plasma Aβ protein levels 77. Intraperitoneal injection of the drug into AD model mice can not only ameliorate cognitive decline but also reduce neuronal apoptosis and Tau protein phosphorylation 78. In addition, a study on AD-related diseases in humans showed that the level of c-Abl in the brains of AD patients was obviously higher than that in the brains of controls. The Abl-py412, an activated form of c-Abl phosphorylated on tyrosine residue 412, has an increased level in the early stage of AD. In the late stage of the disease, the level of abl-py412 is mainly increased in the hippocampal area. The Abl-pt735, an alternative phosphorylated form of c-Abl on threonine residue 735, has an increased level specifically in the hippocampal region. Furthermore, c-Abl and phosphorylated Tau protein interact in the brains of AD patients, and there is a certain correlation between the levels of these two proteins. The above results show that functional state of c-Abl is different at different stages of AD and that phosphorylated c-Abl and Tau protein interact in the pathogenesis of AD 54.

PD is the second most common neurodegenerative disease in the elderly. Studies have found that Parkin is a substrate of c-Abl and that c-Abl can specifically phosphorylate Parkin at Y143 79-80. The main pathway of this disease is the direct phosphorylation of α-synuclein at Y39 by c-Abl, which leads to abnormal aggregation of α-synuclein and a reduction in Parkin expression. In addition, c-Abl can phosphorylate E3 ubiquitin ligase at Y143 and inactivate it 81-82. These changes can lead to an abnormal increase in the level of toxic protein zinc finger protein 746 (PARIS) and aminoacyl tRNA synthetase complex-interacting multifunctional protein 2 (AIMP2) 81-82. In addition, previous studies have demonstrated that virus induced PARIS transgenic mice can lead to c-Abl activity dependent on PD characteristics such as dyskinesia, dopaminergic neuron loss and neuroinflammation 83. Nilotinib, a c-Abl blocker, can significantly reduce c-Abl activity and Parkin levels, and improve neuronal apoptosis and cognitive function 84. Wu et al. 85 found that in the study of BV2 microglia and PD model of mouse brain neuroinflammation induced by lipopolysaccharide (LPS), nilotinib can reduce TNF-α, IL-1β, IL-6, iNOS, COX-2, and other proinflammatory factors in BV2 cells. And it can significantly inhibit LPS induced neuroinflammation. In addition, tyrosine hydroxylase (TH) is an important player in PD, and nilornib can increase the number of TH- and Nissl-positive neurons in PD patients. It has also been found that c-Abl blockers can significantly reduce dopaminergic neuron loss in c-Abl knockout animals 80. *In vitro* studies, ladotinib can protect against mitochondrial function impairment caused by α-synuclein and reduce the formation of α-synuclein inclusions. Furthermore, *in vivo* studies, the drug can effectively reduce dopaminergic neuron loss and neuroinflammation and improve cognitive function 86. The intracellular inflammasome complex is involved in the recognition and execution of host inflammatory responses. Studies using pharmacological inhibition of c-Abl found that dasatinib reduced inflammasome activation, mitochondrial oxidative stress and LPS induced microglial activation 87. Besides, the relate vitro study also indicated that c-Abl mediated microglia activation may be an important source of inflammatory mediators 88. In addition, many studies have shown that the activity and expression of c-Abl are significantly increased in the brains of PD patients. Kinase blockers can improve brain function, reduce neuron death, inhibit CDK5 phosphorylation, regulate α-synuclein elimination, and inhibit Parkin phosphorylation 89-93. In addition to animal experiments, clinical studies have also found that the treatment of patients with moderate or severe PD with nilotinib for 6 months can significantly alleviate cognitive symptoms 94. In addition, some studies have found that c-Abl kinase inhibitor PD180970 can reduce Toll like receptor-4 mediated NF-κB and inhibits the release of pro-inflammatory cytokines such as IL-6 and monocyte chemoattractant protein-1 (MCP-1) 95.

In addition to the above common neurodegenerative diseases, c-Abl is also associated with Niemann-Pick C (NPC), a fatal autosomal recessive disease characterized by the accumulation of free cholesterol and sphingolipids in the endolysosomal system. c-Abl is activated and triggers neuronal apoptosis *in vitro* and *in vivo* nasopharyngeal carcinoma models 96-97. Klein et al. found that the c-Abl/p73 pathway is related to neurodegeneration in NPC and that c-Abl blocker can delay neurodegeneration in this disease 96. In addition, in other studies of NPC models in neurons, the c-Abl/Histone deacetylases (HDAC2) signaling pathway was found to be involved in the regulation of neurons. Inhibition of c-Abl may be a pharmacological strategy for preventing the adverse effects of elevated HDAC2 levels in nasopharyngeal carcinoma patients 98. In addition, c-Abl is involved in ALS, which is characterized by neuron death. In ALS, c-Abl signaling is triggered through mitochondrial alteration-mediated ROS production 99. Some researches suggest that c-Abl is a treatment target for ALS, and it has been found that the c-Abl blocker dasatinib has neuroprotective effects against this disease *in vitro* and *in vivo* 100. Small interfering RNA (siRNA)-mediated c-Abl gene knockout attenuated the production of proinflammatory mediators in LPS induced glial cell culture 101.

## Conclusions

The expression and activation of c-Abl are abnormally elevated in various neurodegenerative diseases (AD, PD, NPC, ALS, etc.), mainly as a result of neuroinflammation, OS, and abnormal Aβ and Tau protein. In addition, various c-Abl blockers (nilotinib, imatinib, dasatinib, ladotinib and other drugs) can effectively reduce abnormal protein levels and ameliorate cognitive dysfunction. The main mechanism of anesthesia-induced POCD is very similar to the mechanism by which c-Abl is involved in the pathogenesis of neurodegenerative diseases. Therefore, we hypothesized that anesthesia-induced POCD may also be related to abnormal activation or increased expression of c-Abl following the exposure to anesthetic drugs. These drugs, which may have certain preventive effects against POCD, may be new options for the treatment of postoperative neurological dysfunction caused by surgery and anesthesia. However, high-quality studies confirming specific role and potential mechanism of c-Abl in dementia and cognitive decline after anesthesia are lacking. c-Abl is a very promising target for perioperative intervention and treatment of POCD.

## Facts

- c-Abl plays a crucial role in neurodegenerative diseases, mainly through mechanisms such as neuroinflammation, oxidative stress (OS) and Tau protein phosphorylation.
- Blockers of c-Abl may have a good preventive and treatment effects on postoperative neurodegeneration.
- This article summarizes the current understanding and research progress on the mechanisms by which c-Abl may be related to postoperative neurodegeneration.

## Open questions

- Does c-Abl inhibition have anti-inflammatory and neuroprotective effects?
- Does c-Abl inhibition alleviate postoperative cognitive dysfunction (POCD)?
- Through which mechanisms is c-Abl related to POCD?

## Abbreviations

ABL: Abelson
c-Abl: Abelson nonreceptor tyrosine kinase
CNS: central nervous system
POCD: postoperative cognitive dysfunction
PD: Parkinson's disease
AD: Alzheimer's disease
CDK5: cyclin-dependent kinase 5
GVD: granulovacuolar degeneration
ATM: ataxia-telangiectasia mutated
NFT: neurofibrillary tangle
PARIS: zinc finger protein 746
AIMP2: aminoacyl tRNA synthetase complex-interacting multifunctional protein type 2
TH: tyrosine hydroxylase
NPC: Niemann-Pick C
ALS: amyotrophic lateral sclerosis
MAPKs: mitogen activated protein kinases
LPS: lipopolysaccharide
MCP-1: monocyte chemoattractant protein-1
siRNA: small interfering RNA
E3: ubiquitin protein ligase
H2O2: hydrogen peroxide
JIP1: c-Jun interacting protein-1
E9: embryos day9
MTL: medial temporal lobe
WML: white matter lesions
